# Paradoxical Neuroethical Crisis of Agency and Identity in an Obsessive-Compulsive Disorder Deep Brain Stimulation Patient

**DOI:** 10.1192/j.eurpsy.2022.1909

**Published:** 2022-09-01

**Authors:** B. Carr

**Affiliations:** University of Florida, Psychiatry, Gainesville, United States of America

## Abstract

**Introduction:**

Deep Brain Stimulation is an increasingly viable, well-established treatment for medication refractory obsessive-compulsive disorder. Yet, its neuromodulatory effects on the brain have led to varying and opposing neuroethical debates about its potential influence on a range of phenomena such as human agency, sense of nonauthenticity and identity.

**Objectives:**

Establish the importance of maintaining the psychotherapeutic alliance in a long-term DBS patient who reported minimal device side effect and no brain-technology interface interpersonal issues; yet struggled with a paradoxical phenomenon of psychic distress surrounding issues of agency and identity, not through device implantation, but through morphology of cognitions from negativistic interpersonal dynamics and spousal victim-blaming due to the necessity for such a device.

**Methods:**

Case-report of a 60+-year-old gentleman with a history of childhood-onset, treatment refractory OCD with a 15-year history of bilateral DBS lead placed via a ventral caudate/ ventral striatum trajectory through the anterior limb of the internal capsule to the nucleus accumbens.

**Results:**

Years later he was only minimally improved above baseline; yet now with a few-years increasing degree of distress over a perceived atrophy of his capabilities that he felt was validated through what he described as his failure of artificial bionics. Extensive device setting re-optimization did not improve efficacy and with supportive therapy, the DBS device was weaned, and turned off.

**Conclusions:**

The following year the therapeutic foci were on interpersonal identity, existential acceptance of breakthrough symptoms, and engagement of spouse into marital counseling leading to subsequent resolution of distress with improved quality of life.

**Disclosure:**

No significant relationships.

